# Seroprevalence of *Leptospira* spp. in Horses in Israel

**DOI:** 10.3390/pathogens10040408

**Published:** 2021-04-01

**Authors:** Sharon Tirosh-Levy, Miri Baum, Gili Schvartz, Boaz Kalir, Oren Pe’er, Anat Shnaiderman-Torban, Michael Bernstein, Shlomo E. Blum, Amir Steinman

**Affiliations:** 1 Koret School of Veterinary Medicine, The Robert H. Smith Faculty of Agriculture, Food and Environment, The Hebrew University of Jerusalem, Rehovot 7610001, Israel; gili.schvartz@mail.huji.ac.il (G.S.); waitzkalir@gmail.com (B.K.); orenpvet@gmail.com (O.P.); anat.shnaiderman@mail.huji.ac.il (A.S.-T.); amirst@savion.huji.ac.il (A.S.); 2 Division of Bacteriology and Mycology, Kimron Veterinary Institute, Bet Dagan 50200, Israel; mirib@moag.gov.il (M.B.); mdbern51@yahoo.com (M.B.); Shlomobl@moag.gov.il (S.E.B.)

**Keywords:** *Leptospira*, leptospirosis, Pomona, horse, equine recurrent uveitis

## Abstract

Leptospirosis has been reported in both humans and animals in Israel but has not been reported in horses. In 2018, an outbreak of *Leptospira* spp. serogroup Pomona was reported in humans and cattle in Israel. In horses, leptospirosis may cause equine recurrent uveitis (ERU). This report describes the first identification of *Leptospira* serogroup Pomona as the probable cause of ERU in horses in Israel, followed by an epidemiological investigation of equine exposure in the area. Serologic exposure to *Leptospira* was determined by microscopic agglutination test (MAT) using eight serovars. In 2017, serovar Pomona was identified in a mare with signs of ERU. Seven of thirteen horses from that farm were seropositive for serogroup Pomona, of which three had signs of ERU. During the same time period, 14/70 horses from three other farms were positive for serogroup Pomona. In 2015, two years prior to this diagnosis, 259 horses from 21 farms were sampled and one horse tested seropositive for serovar Icterohaemorrhagiae. In 2018, one year later, 337 horses were sampled on 29 farms, with none testing seropositive. Although horses are not considered a major host of *Leptospira* spp., it appears that horses may be infected, and clinically affected, in the course of an outbreak in other species. The identification of leptospirosis in stabled horses may impose a significant zoonotic risk to people.

## 1. Introduction

Leptospirosis is a wide-spread zoonotic disease that affects most mammalian species, with considerable public health and veterinary importance. It is caused by the pathogenic species in the genus *Leptospira*, which are classified into genomospecies according to their DNA phylogenetic distance and into serovars according to their antigenic properties [1,2,3,4]. Leptospirosis in humans and animals may cause a wide variety of symptoms. The main route of bacterial spread and source of infection is through the urine of infected animals and contaminated water or soil. Environmental contamination with infective *Leptospira* is maintained by subclinical carriers. Subclinical carriage is more common in host-adapted serovars such as Icterohaemorrhagiae in rats, Canicola in dogs, Bratislava in horses, Hardjo in cattle, and Pomona in pigs [2,3].

In horses, leptospirosis is considered relatively uncommon, however, its incidence and importance remain uncertain. As in other animals, most epidemiological studies in horses are based on serological evidence of infections. Seroprevalence and main serovars affecting horses vary greatly between studies and regions [5]. The characteristic clinical manifestations of leptospirosis have been described in foals, whereas in adult horses, leptospirosis is mainly associated with late-term abortions and equine recurrent uveitis (ERU) [5]. ERU is a common sequela of *Leptospira* infection in horses, which usually develops 12–24 months after exposure [5,6,7,8]. *Leptospira* spp. is considered the most common infectious cause of ERU in Europe and North America [5,9,10].

In Israel, up to 70 seropositive animals—mainly cattle, dogs, and pigs—have been reported annually until 2018 (https://www.moag.gov.il/vet/dochot-shnatiim/Pages/default.aspx accessed on 9 January 2021). Human leptospirosis is rarely reported, with 5–10 annual cases, most of which are travel-related [11,12,13]. In 2018, an unusual outbreak of human leptospirosis was reported, with approximately 500 documented cases. This outbreak was associated with water recreational activities in specific rivers at the Galilee Lake rivers basin [14]. This outbreak was followed by an increased prevalence of animal leptospirosis in the same region through 2019. The 2018 human outbreak also prompted an epidemiological investigation that revealed high seroprevalence in beef cattle and wild boars in northern Israel (https://www.moag.gov.il/vet/dochot-shnatiim/Pages/default.aspx accessed on 9 January 2021). Since its first detection in a dairy farm in 2002, and reoccurrence in a beef feedlot in 2010, serogroup Pomona has become the main *Leptospira* spp. serogroup detected in cattle in Israel and has spread over most of the country.

Clinical leptospirosis has not been previously documented in horses in Israel. Since 2004, two hundred and six equine sera were tested for leptospirosis in the Kimron Veterinary Institute (https://www.moag.gov.il/vet/dochot-shnatiim/Pages/default.aspx accessed on 9 January 2021), with no recorded positive tests until 2017. This manuscript presents the first report of clinical leptospirosis in horses in Israel and the following epidemiological investigation describing the prevalence of *Leptospira* spp. serovars in the Israeli equine population.

## 2. Results

### 2.1. Investigation of Leptospira spp. Seroprevalence and the Occurrence of ERU Cases in One Farm

A 13 years-old Paint horse mare was clinically suspected as a case of ERU in October 2017 by the referring veterinarian. Since there was no improvement after two months of topical treatment, the mare was referred for further evaluation and a serum sample was sent to test for *Leptospira* spp. The mare tested seropositive for serogroup Pomona, with an antibody titer of 400 (#6 in Table 1).

**Figure 1:**
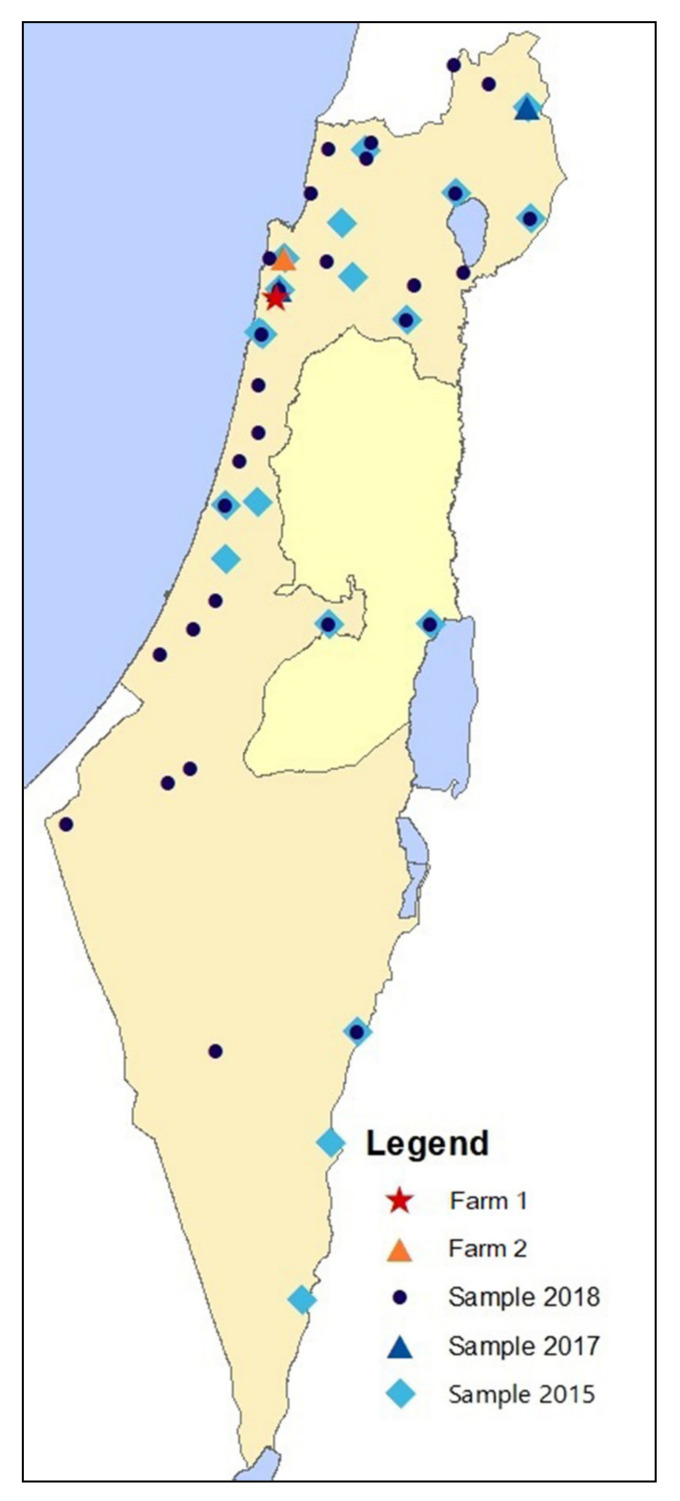
The geographical distribution of the farms sampled in 2015 (light blue diamonds), in 2017 (blue triangles), and in 2018 (dark blue circles). Farms in which seropositive horses for *Leptospira* spp. serogroup Pomona were detected in 2017 are marked with a red star (farm 1) and an orange triangle (farm 2).

The mare resided in a farm (farm 1) located in the Carmel area, along with 12 additional horses (Figure 1). The farm was a riding school that consisted of a shed with a tack room and stalls, but the horses were mostly turned out to a small pasture behind the farm, bordering a large cattle grazing area occupied mainly by beef cattle, but frequently exposed to wild animals such as rodents, foxes, jackals, hyenas, and wild boars.

Of the 13 horses sampled, seven were seropositive for *Leptospira* spp. serogroup Pomona, while several also showed reactions to other *Leptospira* serovars (Table 1). None of the horses were new to the farm and all were considered healthy, according to the owner. Two additional horses had a suspected clinical history of ERU in the last two years, with progressively deteriorating vision (horses #11 and #13 in Table 1), and another mare developed ERU six months following this investigation (#1 in Table 1); all three tested seropositive for serogroup Pomona, the latter with significantly high antibody titer (1600).

Following the detection of seropositive horses, the farm was quarantined for two weeks according to the instructions of the Israeli veterinary services, and all horses were treated with enrofloxacin (7.5 mg/kg, orally, once daily, Baytril, Bayer, Germany) for ten days. The choice of antimicrobial agent was based on its availability as an oral drug and its documented ocular penetration in horses [15].

Ten months after the initial investigation, the farm was revisited, and all available horses were re-tested. All three horses that initially suffered from ERU had been sold, as their impaired vision prevented their use as school horses. One additional horse was brought to the farm (#14 in Table 1). All remaining horses that were seropositive in 2017 were seropositive in 2018, with lower or similar antibody titers. None of the horses seroconverted between 2017 and 2018 (Table 1).

### 2.2. Sero-Epidemiological Survey of Equine Exposure to Leptospira spp. in Israel

In 2015, two years prior to the documented cases, none of 259 horses from 21 farms throughout Israel were seropositive for *Leptospira* spp. serogroup Pomona. Only one horse tested seropositive for serovar Icterohaemorrhagiae, and five additional horses had suspect titers to serovars Icterohaemorrhagiae (*N* = 3), *L.* Hardjo (*N* = 1), and *L.* Canicola (*N* = 1) (Table 2, Figure 1).

In August 2017, four months prior to the diagnosis of *Leptospira* seropositive horses in farm 1, 70 horses were sampled on three farms, including two in the Carmel area, near this farm. Fourteen horses tested seropositive and seven were suspected of exposure to serogroup Pomona, all from one farm (farm 2) located 17.5 km from farm 1 (Figure 1). Sixteen of the seropositive or suspect horses were also sampled during 2015 and were negative for all serovars. Of the seropositive or suspected horses for serogroup Pomona, three also tested seropositive and six were suspected for serovar Ballum. One additional horse from that farm and another from a farm in the Golan Heights (80 km from farm 1) were suspected of exposure to serovar Ballum. No seropositive horses were detected in the farm located 3.5 km from the outbreak farm (Table 2, Figure 1).

Farm 2 was a holding of approximately 40 horses that were kept in pasture and bred mainly Appaloosa horses. During a follow-up call on May 2020, the caretakers of the horses on that farm reported increased occurrence of ocular disease on the farm, including five horses that had become blind in one or both eyes since 2018. Of the ten horses with reported ocular lesions in the last two years, five tested seropositive and three were suspected seropositive for *Leptospira* spp. serogroup Pomona in 2017.

In 2018, nine to ten months after the first reported case on farm 1, 337 horses were sampled on 29 farms and none tested seropositive for *Leptospira* spp. serogroup Pomona. One horse had a suspect titer to serovar Canicola and one had a suspect titer to Bratislava. This sample included two farms located in the Carmel area, but not farm 2 (Table 2, Figure 1). The second screening for farm 1 was conducted during the same timeframe, and four of ten horses remained seropositive for serogroup Pomona.

## 3. Discussion

This is the first description of ERU possibly related to leptospirosis in horses in Israel, which initiated a detailed epidemiological investigation to better understand its scope. Although *Leptospira* spp. is one of the main causes of ERU in horses worldwide, as well as a cause of abortion and systemic disease [5,6,7,10,16,17], it was not considered a significant cause of ERU in the differential diagnosis list (DD) to most Israeli equine practitioners. Although the causative agent was not isolated or directly identified by PCR, the diagnosis of exposure to *Leptospira* spp. in the cluster of ERU cases highlights the importance of including this pathogen in the DD of suspected clinical cases.

All ERU cases in this investigation reacted against serogroup Pomona in the serological exam. Pomona is the main serogroup associated with ERU and equine abortions in North America [9,17,18,19,20]. It has also been identified in horses in Italy [21,22], although the predominant serovar associated with ERU in Europe is Grippotyphosa [10,16,17].

Exposure to *Leptospira* spp. serogroup Pomona does not appear to have been common in horses in Israel. No seropositive horses for this serogroup were identified in 40 farms distributed throughout the country in 2015 and in 2018. Horses seropositive for Pomona were only identified in two farms located in the Carmel area. Unfortunately, only one of these farms (farm 2) was sampled in 2015, and no data was available on farm 1 prior to the identification of the suspected clinical cases. In both positive farms (Farms 1 and 2, 17.5 km apart) horses are used for horseback riding and are kept in paddocks or pastures and, therefore, may be exposed to a variety of wild and peri-domestic animal species, including beef cattle and wild boar. Cases of ERU have been reported in both farms since the documented exposure to serogroup Pomona, and several horses were removed from both farms after developing severely impaired vision. According to caretakers of both farms, none of the horses exhibited any systemic signs of disease that might be attributed specifically to *Leptospira* infection. A young horse (1.5 years, #7 in Table 1) from farm 1 was diagnosed with acute kidney injury (AKI) and euthanized four weeks after the first detection of *Leptospira* spp. serogroup Pomona in the farm. This horse tested negative for *Leptospira* spp. both in the initial survey of the farm and during his hospitalization, however, samples may have been taken in early stages of infection, before specific antibodies could have been detected. Necropsy examination was inconclusive regarding the initial cause of kidney damage.

Serological evidence from active and passive surveillance has shown that serogroup Pomona has spread in Israel in the last decade, and that grazing beef cattle and wild boars may be probable sources of spread and maintenance of this pathogen in the environment (SEB, personal communication). During the survey in 2015, there was already serological evidence for the presence of serogroup Pomona in southern Israel, however, none of the horses in this study tested positive at that time point. This may be attributed to the fact that none of the horse farms tested in the south had close proximity to cattle grazing areas, or to the differences in climate, available pasture areas, and prevalent wildlife species between the north and the south of the country. The geographic and temporal progression reported in cattle reached the Carmel area in 2015–2018 (SEB, personal communication), which corresponds with the detection of seropositive equines, and later resulted in an outbreak of human cases in the Golan Heights (approximately 80 km northeast) in 2018 [14]. The documented seroconversion of horses in farm 2 between 2015 and 2017 suggests that the exposure to this pathogen was novel in this area. The lack of seroconversion in horses in farm 1 between 2017 and 2018 could indicate that the source of exposure may have been eliminated; however, additional future surveillance is required to evaluate the presence and spread of this pathogen.

Exposure of horses to other *Leptospira* serovars was surprisingly low. In 2015 only one horse (1/259, 0.4%) tested seropositive for serovar Icterohaemorrhagiae and none tested seropositive in 2018. In 2017, all four seropositive horses for serovar Ballum were also seropositive for serogroup Pomona, which may suggest exposure to multiple Leptospirae or serological cross-reactivity, as was suggested previously [22]. Both these serovars (Icterohaemorrhagiae and Ballum) are maintained mainly by rodents [23]. In farm 1, three of the seven horses seropositive for serogroup Pomona also tested positive for serovars Bratislava and/or Icterohaemorrhagiae, however, only one horse (horse #12 in Table 1) had higher serological titer for serovar Bratislava than for serovar Pomona. The fact that the overall exposure of horses to all serovars in 2015 was very low, whereas in 2017 there was increased prevalence of serogroup Pomona in horses and other animals, and that overall higher titers were found for serovar Pomona in the same farm, support the identification of serovar Pomona as the main source of exposure in most positive horses.

This study was based on seroprevalence and as such should be interpreted accordingly. The MAT is highly specific for *Leptospira* and, therefore, it provides strong evidence of the prevalence of *Leptospira* exposure, up to the serogroup level. In future works, pathogen detection by PCR and culture should be investigated to identify the actual serovars and genotypes present in Israel.

## 4. Materials and Methods

### 4.1. Study Design

After serological detection of *Leptospira* from a clinical case of ERU in 2017, an investigation was conducted on the infected farm (farm 1) (Figure 1). The medical history was recorded, and serum samples were collected from all horses (*N* = 13) and two dogs that resided on the farm. Following the identification of several seropositive horses on that farm, we aimed to determine the seroprevalence of horses before and after 2017. To this effect, we used sera that had been collected in the winter of 2015, in which 259 horses were sampled on 21 farms, and sera that had been collected in the summer of 2018, in which 347 horses were sampled on 30 farms (Figure 1). The 2018 population included nine of the farms that were sampled during 2015 and the farm in which clinical cases were detected in 2017 (farm 1). Both surveys were designed to reflect the geographical distribution of the Israeli horse population, and all animals sampled were apparently healthy and did not demonstrate any clinical signs of disease. Three additional farms from the 2015 survey were also sampled during 2017 (one of which, farm 3, was located approximately 3.5 km from farm 1 and was sampled on all three occasions). None of the horses was vaccinated against leptospirosis, and such vaccine is not available in Israel for horses.

### 4.2. Sample Collection

Blood was collected from the jugular vein of all horses and the cephalic vein of the dogs into sterile vacuum tubes without an anticoagulant. Sera were obtained from the clotted blood samples by centrifugation (4000× *g* for 10 min) and kept at −20 °C until processing.

Sample collection was performed under the horse owners’ consent, and the study was approved by the Internal Research Committee of the Koret School of Veterinary Medicine—Veterinary Teaching Hospital (KSVM-VTH/23_2014, KSVM-VTH/02_2018).

### 4.3. Serological Screening

All sera were screened for the presence of anti-*Leptospira* antibodies using the MAT against eight serovars: Ballum, Bratislava, Canicola, Grippotyphosa, Hardjo, Icterohaemorrhagiae, Pomona, and Tarassovi [24]. Antigens of the above serovars were grown in supplemented EMJH (Ellinghausen–McCullough–Johnson–Harris) medium (BD Difco, Sparks, MD, USA) for about five days up to turbidity equivalent to McFarland 1 standard. Antigens quality control included visual macroscopic and dark-field microscopic inspection for contaminants, and tests against immune-specific sera (AMC, Amsterdam, Netherlands). Prior to testing, frozen sera were completely thawed and homogenized. Sera were diluted 1:25 in formalized phosphate buffered saline (PBS), and diluted two-fold thereafter. Volumes of 0.025 mL of diluted sera were added to the same volume of antigen culture in flat microtiter plates and incubated at room temperature overnight. Plates were examined by dark-field microscopy for agglutination. Test results with titers 200 or higher were considered “positive”, while titers of 100 were considered “suspect” and tests with titers 50 or lower were considered “negative”.

## 5. Conclusions

This is the first report of exposure to *Leptospira* spp. in horses in Israel, demonstrating generally low exposure to environmental strains with occasional exposure to more pathogenic serovars, which may affect both equine and human health. The description of clinical cases possibly related to serogroup Pomona in two farms from the same geographical region corresponded with documented cases in other species in the same area and timeframe, indicating possible zoonotic risk. This report implies that leptospirosis must be considered a possible cause of ERU in horses in the area. Serological identification of exposed animals can create the basis for initial epidemiological investigation, but continued emphasis should be given to the sampling of animals during the acute phase in order to allow for the genotypic characterization of the *Leptospira* strains affecting horses in Israel by direct molecular methods or bacterial isolation. Although not yet supported by the data from 2019, hopefully, the introduction in 2017–2018 of compulsory vaccination of beef cattle in areas deemed to pose a public health risk will have its effect in reducing the environmental contamination and spread of the disease.

## Figures and Tables

**Table 1:** Serological screening for *Leptospira* exposure in animals from Farm 1, Carmel, Israel, during 2017 and 2018.

Animal	Sex	Breed	Age (Years)	Lepto 8	Ballum	Bratislava	Canicola	Grippotyphosa	Hardjo	Icterohaemorrhagiae	Pomona	Tarassovi	ERU	Pomona 2018
Horse 1	F	M	11	+	100	800	50	50	<50	400	1600	<50	6 months later	800
Horse 2	M	PP	9	+	<50	100	<50	<50	<50	100	400	<50		400
Horse 3	M	QH/TW	3	-										<50
Horse 4	M	M	8	+	<50	50	<50	<50	<50	100	200	<50		200
Horse 5	F	QH	14	-										<50
Horse 6	F	PH	13	+	<50	100	<50	<50	<50	100	400	<50	+	na
Horse 7	M	PH	1.5	-										na
Horse 8	M	M	22	-										<50
Horse 9	F	M/TB	32	-										<50
Horse 10	M	PH	8	-										<50
Horse 11	M	Sh	25	+	<50	50	<50	<50	50	50	800	<50	+	na
Horse 12	M	Ar	12	+	<50	400	<50	<50	<50	100	200	<50		200
Horse 13	M	M/QH	21	+	<50	200	<50	<50	<50	400	800	<50	+	na
Horse 14	M	QH	4	na										<50
Dog 1	M		15	-										
Dog 2	M		2	-										

Serological screening was performed using microscopic agglutination test (MAT) against eight serovars in 2017 and against serogroup Pomona in 2018. Antibody titers are specified for each positive serovar. Titers of 200 or higher were considered positive. Lepto 8: initial screening for all eight serovars; na: not available; +: seropositive for leptospirosis; -: seronegative for leptospirosis. Sex: M: male; F: female. Breed: M: mixed; Ar: Arabian; PH: Paint Horse; PP: Peruvian Paso; Sh: Shire; QH: Quarter Horse; TB: Thoroughbred; TW: Tennessee Walking Horse. ERU: +: clinical signs consistent with equine recurrent uveitis. Pomona 2018: repeated serological screening for serogroup Pomona in 2018.

**Table 2:** Equine serologic exposure to *Leptospira* serogroup Pomona in Israel in 2015, 2017, and 2018.

Farm Location	X	Y	*N* 2015	Pomona 2015	*N* 2017	Pomona 2017	*N* 2018	Pomona 2018
Ofer (Farm 1)	32.622500	34.982204			13	7	10	4
Beit Oren (Farm 2)	32.733303	35.013272	18	0	33	14		
Kerem Maharal (Farm 3)	32.646959	34.992953	18	0	8	0	8	0
Abirim	33.040550	35.284438					4	0
Alonim	32.721899	35.143353					13	0
Beer Tuvia	31.733619	34.724240					15	0
Beit Hanania	32.529429	34.926170	4	0				
Beit Zera	32.689282	35.573251					5	0
Binyamina	32.519224	34.949134	7	0			15	0
Brechia	31.667969	34.622417					10	0
Cabri	33.024736	35.149709					21	0
Ein Dor	32.656064	35.419218					6	0
Ein HaMifratz	32.903858	35.097995					8	0
Ein Harod	32.563529	35.392509	9	0			9	0
Ein Vered	32.262046	34.927822					7	0
Ein Yahav	30.671991	35.240339	5	0			4	0
Gilat	31.327564	34.649024					14	0
Givaat Haim	32.392304	34.929899					13	0
Grofit	29.941640	35.066205	17	0				
Iblin	32.827889	35.184706	7	0				
Ifat	32.675016	35.225774	31	0				
Kalia	31.750493	35.466092	7	0			8	0
Kefar Sauld	33.194325	35.658630					7	0
Kefar Sirkin	32.074643	34.926689	12	0				
Kefar Truman	31.981771	34.922963	20	0				
Kefer Veradim	32.996595	35.256156					10	0
Kidron	31.821931	34.796826					16	0
Ma’aona	33.019701	35.258907	11	0				
Megadim	32.730275	34.956857					8	0
Merom Golan	33.133944	35.777086	27	0	29	0		
Misgav Am	33.247662	35.548156					7	0
Mishmar HaNegev	31.364297	34.717401					5	0
Mizpe Ramon	30.627905	34.807843					25	0
Naan	31.883792	34.858009	5	0				
Nezer Sireni	31.921826	34.821859	15	0				
Nov	32.831904	35.783060	8	0			7	0
Ora	31.755119	35.153482	9	0			8	0
Paran	30.362050	35.156747	4	0				
Raanana	32.184838	34.871028					20	0
Ramat Gan	32.068449	34.826131	7	0			32	0
Sde Avraham	31.210914	34.337020					10	0
Vered Hagalil	32.904466	35.551128	18	0			22	0

The geographic location (coordinates X and Y), the number of horses screened (*N*), and the number of seropositive horses are specified for each sampling date.

## Data Availability

Data is contained within the article.
